# Characteristics of highly impaired children with severe chronic pain: a 5-year retrospective study on 2249 pediatric pain patients

**DOI:** 10.1186/1471-2431-12-54

**Published:** 2012-05-16

**Authors:** Boris Zernikow, Julia Wager, Tanja Hechler, Carola Hasan, Uta Rohr, Michael Dobe, Adrian Meyer, Bettina Hübner-Möhler, Christine Wamsler, Markus Blankenburg

**Affiliations:** 1 German Paediatric Pain Centre and Vodafone Foundation Chair of Children’s Pain Therapy and Paediatric Palliative Care, Children’s and Adolescents’ Hospital, Datteln, Witten/Herdecke University, Faculty of Health – School of Medicine, Dr.-Friedrich-Steiner Str. 5, Datteln, 45711, Germany

**Keywords:** Children, Chronic pain, Impairment, Risk factors, Pediatric

## Abstract

**Background:**

Prevalence of pain as a recurrent symptom in children is known to be high, but little is known about children with high impairment from chronic pain seeking specialized treatment. The purpose of this study was the precise description of children with high impairment from chronic pain referred to the German Paediatric Pain Centre over a 5-year period.

****Methods**:**

Demographic variables, pain characteristics and psychometric measures were assessed at the first evaluation. Subgroup analysis for sex, age and pain location was conducted and multivariate logistic regression applied to identify parameters associated with extremely high impairment.

**Results:**

The retrospective study consisted of 2249 children assessed at the first evaluation. Tension type headache (48%), migraine (43%) and functional abdominal pain (11%) were the most common diagnoses with a high rate of co-occurrence; 18% had some form of musculoskeletal pain disease. Irrespective of pain location, chronic pain disorder with somatic and psychological factors was diagnosed frequently (43%). 55% of the children suffered from more than one distinct pain diagnosis. Clinically significant depression and general anxiety scores were expressed by 24% and 19% of the patients, respectively. Girls over the age of 13 were more likely to seek tertiary treatment compared to boys. Nearly half of children suffered from daily or constant pain with a mean pain value of 6/10. Extremely high pain-related impairment, operationalized as a comprehensive measure of pain duration, frequency, intensity, pain-related school absence and disability, was associated with older age, multiple locations of pain, increased depression and prior hospital stays. 43% of the children taking analgesics had no indication for pharmacological treatment.

**Conclusion:**

Children with chronic pain are a diagnostic and therapeutic challenge as they often have two or more different pain diagnoses, are prone to misuse of analgesics and are severely impaired. They are at increased risk for developmental stagnation. Adequate treatment and referral are essential to interrupt progression of the chronic pain process into adulthood.

## Background

Chronic pain is a frequent problem not only in adults but also in children and adolescents [1,2]. Epidemiological studies have played an important role in outlining the prevalence and impact of pediatric chronic pain in the general population [3]‐[6] with rates of a 3-month prevalence ranging between 25% and 46% [3,4].

These large epidemiology based studies often define chronic pain solely on the duration of pain, usually >3 months, and have also been hampered by assigning diagnoses according to self-report or pain description in questionnaires. In children with clinically significant impairment from chronic pain, results from these epidemiological studies have limited applicability as they primarily report on the symptom of chronic pain, which may not necessarily cause impairment and, even require treatment. A study in school children has shown that symptoms in many cases resolve spontaneously without treatment [7] and are not necessarily associated with disability, distress and illness beliefs. These features are specific to clinically relevant chronic pain [8], elevating it into the realm of a chronic disease. Second, epidemiological studies include a very small number of highly impaired children. The study by Huguet and Miro [5] investigated a school sample of 561 children with 37% of the children classified as experiencing chronic pain. However, only 1% (n = 6) actually reported a high pain-related disability. Finally, children from epidemiological studies are often recruited in schools [3]‐[5,9], but children with a high level of impairment from chronic pain miss a significant amount of school [10] making it entirely possible they would be underrepresented in epidemiological school samples.

Due to these restrictions, results of epidemiological studies cannot be readily generalized to children suffering significant impairment from chronic pain and who are in need of treatment.

Published pediatric clinical studies, on the other hand, have had very small sample sizes or focused on specific pain problems or age groups [11]‐[15]. Until now, there has not been a description of a large patient sample with well-defined chronic pain conditions classified according to diagnostic criteria with a high level of impairment requiring tertiary pain treatment. More information on the characteristics of these children has the potential to inform treatment conceptualization and implementation of tertiary treatment programs [15,16]. It is also known that health care structures for specialized treatment in highly impaired children with chronic pain are scarce [1,2,17]. A study on the services offered by multidisciplinary pain treatment facilities for children and adolescents with chronic pain across Canada demonstrated the discrepancy between services offered and needed with the result being limited access for children in need of treatment [2]. By improving specialized treatment and access to this treatment poor developmental and functional outcomes may be prevented [15,18].

In epidemiological studies, some factors seem to be highly correlated with the degree of the overall impairment due to chronic pain. Huguet and Miro [5] showed that children with headache experience severe pain along with high pain-related disability more often compared to other pain conditions. They also report that younger children experience less severe pain and a non-specific effect of sex on pain severity. Physical and psychosocial functioning was found to decrease with increasing pain severity [5]. Other clinical studies report on the negative effect of depression on school attendance in chronic pain patients [19], or the relation of anxiety and pain related-disability mediated by passive pain coping [20]. Finally, pain severity was also found to be related to previous treatment and medication use in a sample of school children [5]. In this study, impairment was operationalized as a comprehensive measure based on pain duration, pain frequency and intensity, pain-related school absence and disability in every-day life [18,21].

The main objectives of this study were to detail the characteristics of children and adolescents with chronic pain seeking tertiary care and identify factors associated with extremely high impairment. In so doing, it is anticipated that insights will be gained into the requirements of the health care system to deliver services for this vulnerable group of children and adolescents.

## Methods

### Sample

All children who completed the standard initial 1.5-hour evaluation at the German Paediatric Pain Centre over the five year period July 1st 2005 to June 30th 2010 were included in the study. Patients were referred by their primary pediatrician or pediatric hospital due to chronic pain.

### Tertiary pediatric pain clinic

The German Paediatric Pain Centre, a tertiary pediatric pain clinic with expertise in treating a wide range of chronic pain conditions, offers a multimodal and multidisciplinary treatment for children with chronic pain in either an inpatient or outpatient setting. Children with life-limiting or life-threatening conditions are not referred to the pain clinic but are seen by the pediatric palliative service affiliated to the center.

The staff at the center are highly experienced in pediatric pain management and child psychotherapy with, on average, four years clinical experience. The center has an active quality maintenance program with daily supervision of each case by the head of the department (BZ) or his assistant (CH), weekly multi-professional team discussions as well as monthly pain conferences with external experts.

Each new child and their family have an initial 1.5-hour evaluation by a pediatrician, child psychologist and pediatric nurse [22]. The key goals of this session are to identify the nature of the chronic pain experience, educate the child and their parents on the biopsychosocial model of chronic pain, provide appropriate treatment recommendations and prevent further diagnostic or medical interventions that are not indicated. ICD-10 based pain diagnoses are given and according to clinical guidelines one of two different treatment recommendations is assigned based on required treatment intensity: multimodal outpatient treatment [22] or an intensive multimodal inpatient treatment [18,21,23].

### Procedure

Children and their parents were mailed a battery of questionnaires prior to the first appointment. The questionnaires sent were the German Pain Questionnaire for Children and Adolescents [24] with the Paediatric Pain Disability Index [25], to assess demographic and anamnestic parameters as well as pain characteristics, the Anxiety Questionnaire for Pupils (Angstfragebogen für Schüler [26]) and the Depression Inventory for Children and Adolescents (Depressionsinventar für Kinder und Jugendliche [27]) to assess emotional distress.

After the initial appointment a clinical letter was written. This included diagnoses, treatment recommendations, and a summary of the most relevant questionnaire information. Data for this study was gathered retrospectively from the clinical letters with further data from the questionnaires obtained from the patient’s chart, if required.

### Measures

#### Demographic information

Sex and age were assessed with the German Pain Questionnaire for Children and Adolescents [24]. Children were assigned to one of five age groups. These groups were created based on developmental and social criteria in Germany. Children aged up to three years are infants and toddlers (0–3y); from the age of four to six the German child attends kindergarten (4–6y); this time is considered as early childhood. After this phase the child enters primary school, middle childhood (7–10y), and then transfers to secondary school. Adolescents (age 11y and older) were divided into two groups (11–14y; ≥15y).

#### Pain characteristics

*Pain location* was assessed by self-report using an illustration of a body, which is part of the German Pain Questionnaire for Children and Adolescents [24]. Children were instructed to mark all pain locations with a cross and, additionally, highlight the main pain location (head, abdomen, back/ extremities, others) with a circle. Children were also separated with regards to the number of pain locations – up to 2 vs. 3 or more.

The *duration of the pain problem* was computed as the time in month, from the time when the current pain problem began to the first appointment at the pain clinic. *Pain frequency* was rated on a scale with 4 different categories from “constant pain” to “less than several times a week”. The *maximal and average pain intensity* in the last four weeks was reported on a numeric rating scale (NRS; with 0 = no pain to 10 = maximal pain).

In children aged 11 years and older duration of the pain problem, pain frequency and pain intensity in the past 4 weeks was measured by self-report while the information was gained by parent proxy report for children younger than 11 years of age.

#### Pain-related disability

Pain-related disability in daily life was assessed by use of the validated German Paediatric Pain Disability Index (P-PDI) [25]. This 12 item questionnaire is used for children aged 11 years and above, while parents report on children younger than 11 years. The questionnaire has good internal consistency (Cronbach’s alpha = .87) and good convergent validity [25].

#### School absence

Parents reported how many days their child missed school within the preceding 20 school days [24]. It has been shown previously that there is a strong association between parent reports on school absence and official school attendance records [10].

#### Emotional distress

The Anxiety Questionnaire for Pupils [26] and the Depression Inventory for Children and Adolescents [27] were used to assess emotional distress.

The Anxiety Questionnaire measures fear of exam, general anxiety and school aversion in children aged 9 years and older. It demonstrates good reliability (Cronbach’s alpha of the scales ranging from .67 to .85) and validity [26]. The Depression Inventory is a self-report measure for children aged 8 years and older that assesses symptoms of depression. It consists of 26 items and has good reliability (Cronbach’s alpha = .84) as well as good discriminant and convergent validity [27].

Norm scores based on a German community sample (standardized T-scores with a mean of 50 and a standard deviation of 10) are available for both measures of emotional distress and for each scale a cut-off T-value of 60 was defined as indicative of increased anxiety and depression, respectively.

#### Health care utilization

Parents reported the number of previous pain-motivated hospital stays and the number of previous physician consultations and diagnostic investigations due to the current pain problem [24]. They also reported analgesics taken within the previous 3 months (current analgesic treatment) and prior analgesic usage (previous analgesic treatment).

#### Impairment

Children with extremely high impairment were identified based on criteria for inpatient admission presented in previous studies [18,21]. Assignment to the extremely high impairment group (inpatient treatment) required 3 of the following 5 criteria to be fulfilled: pain duration ≥ 6 months, constant pain with an average pain intensity of NRS ≥5/10, pain peaks of NRS ≥8/10, at least one week absence from school within the preceding 4 weeks and Paediatric Pain Disability Index ≥36/60 [18,21].

Missing values in the child and adolescent questionnaires for pain characteristics (pain location, pain intensity, pain frequency) were assigned using parents’ proxy report. Some missing values could not be replaced therefore the sample size differs for analyses from n = 1353 to n = 2248.

### Ethics

The study was approved by the local ethics committee of the University hospital. All children and their parents had given prior written informed consent for data collection, electronic data storage and data analysis when they first visited the German Paediatric Pain Centre.

### Statistics

For the detailed depiction of the clinical presentation, group comparisons (sex, age groups, main pain locations) were calculated for different pain parameters by using *Chi*^*2*^-tests, two sample Student’s *t*-tests or analysis of variance (ANOVA). Scheffé tests were applied for multiple post-hoc comparisons. To compare emotional distress data to norm data *Chi^*2*^-*tests or one-sample *t*-tests were performed.

Effect sizes were specified for all significant group comparisons. The measure used for ANOVA results was *eta^*2*^* (>0.01 = small; >0.06 = medium; >0.14 = large effect [28]). The effect size measure calculated for Chi^*2*^-tests was Cramer’s V (>0.1 = small; >0.3 = medium; >0.5 = large effect [28]).

Stepwise logistic regression was calculated to estimate Odds Ratio (OR) and the 95% confidence interval (CI) for variables associated with extremely high impairment, i.e. age (child (<11 years) / adolescent (≥11 years), sex (male / female), pain location (for each pain location: y/n), number of pain locations (<2 / >2), previous hospital stays (y/n), anxiety (normal / increased score), and depression (normal / increased score). All these variables were dichotomized to make interpretation more explicit.

A two-tailed significance level of *p* = .05 was defined as significant. All analyses were calculated using SPSS Version 19.0.

## Results

### Sample characteristics

**Figure 1:**
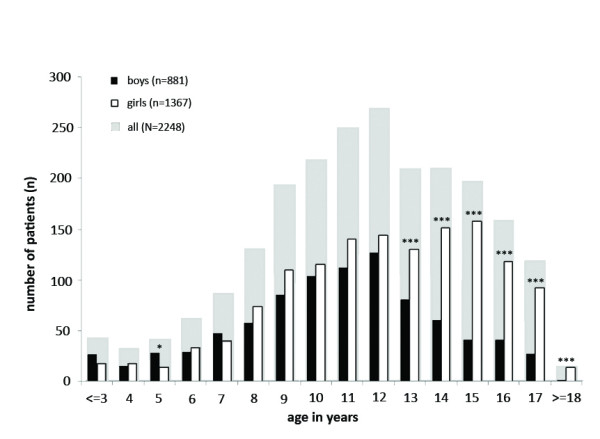
**Distribution of children with chronic pain by age and gender.***Note*. *Chi^*2*^*-test: *** *p* < 0.001; * *p* < 0.05.

Over the 5-year period, July 2005 to June 2010, 2249 children with chronic pain presented for the initial session at the German Paediatric Pain Centre. The majority of children were female (61%); mean age was 11.5 years (*SD* = 3.42). Significantly more girls entered treatment from the age of 13 years onwards (Figure 1).

The primary location of chronic pain for most children was the head, followed by the abdomen then back/extremities (Table 1). The exceptions to this were children <4 years of age who mostly had musculoskeletal pain followed by headache, and the 4 to 6 year and >14 year age groups who have musculoskeletal pain as the second most prevalent location (*Chi^*2*^*(12) = 109.738; *p* < 0.001; Cramer’s V = 0.1). Distribution of main pain location did not differ between boys and girls across all age groups except in 11 to 14 year olds. In this group, girls had more musculoskeletal pain (*Chi^*2*^*(3) = 10.716; *p*  < 0.01; Cramer’s V = 0.1). Eighteen per cent of the sample reported more than two pain locations and more girls 11 years and older reported this finding (*Chi^*2*^(*1) = 19.194; *p* < 0.001; Cramer’s V = 0.1).

**Table 1:** Main location of pain

**Main pain location**	**Total sample**		**0–3 years**		**4–6 years**		**7–10 years**		**11–14 years**		**≥15 years**		**Girls**		**Boys**	
	**n**	**%**	**n**	**%**	**n**	**%**	**n**	**%**	**n**	**%**	**n**	**%**	**n**	**%**	**n**	**%**
Head	**1541**	**69.0**	12	30.0	94	69.1	465	73.8	644	68.7	326	66.8	923	68.0	618	70.5
Abdomen	**364**	**16.3**	3	7.5	18	13.2	102	16.2	176	18.8	65	13.3	210	15.5	154	17.6
Back / extremities	**295**	**13.2**	23	57.5	24	17.6	56	8.9	106	11.4	85	17.4	201	14.8	94	10.8
Other^*^	**32**	**1.4**	2	5.0	-	-	7	1.1	11	1.2	12	2.5	23	1.7	9	1.0
**Total**	**2232**	**100**	**40**	100	**136**	100	**630**	100	**938**	100	**488**	100	**1357**	100	**876**	100
Number of pain locations > 2	**409**	**18.2**	14	35.9	22	16.2	116	18.4	148	15.8	109	22.2	288	21.2	121	13.8

^*^ e.g. whole-body, genital.

Table 2 provides an overview of the diagnoses given after the initial assessment. Common pain diagnoses were tension type headache (TTH) (n = 1071), migraine (n = 956), functional abdominal pain (n = 253), and some form of musculoskeletal pain (n = 408). Irrespective of pain location many children were diagnosed with “*Chronic pain disorder with somatic and psychological factors”* (n = 969) (German ICD-10 diagnosis F45.41).

**Table 2:** Pain diagnoses

	**n**	% (total sample)*	
**Headache diagnosis (n = 1676)**		**74.5**	
Migraine	956	42.5	
Migraine with aura	157	7.0	
Tension type headache (TTH)	1071	47.6	
Secondary Headache^†^	82	3.6	
Trigeminal Neuralgia	9	0.4	
Cranial Neuralgia	6	0.3	
Chronic pain disorder with somatic and psychological factors (Pain location head)	542	24.1	
Others^‡^	81	3.6	
**Abdominal pain diagnosis (n = 501)**		**22.3**	
Functional Abdominal Pain	253	11.2	
Chronic pain disorder with somatic and psychological factors (Pain location abdomen)	204	9.1	
Abdominal pain-related Functional Gastrointestinal Disorders other than functional abdominal pain	27	1.3	
Other^§^	17	0.7	
**Musculoskeletal pain diagnosis (n = 408)**		**18.1**	
Chronic pain disorder with somatic and psychological factors (Pain location extremities)	179	7.9	
Growing Pain	51	2.3	
Complex Regional Pain Syndrome or other peripheral neuropathic pain	58	2.6	
Chronic pain disorder with somatic and psychological factors (Pain location back)	81	3.6	
Other back Pain^||^	54	2.4	
Other musculoskeletal pain^¶^	89	4.0	
**Mixed Pain Diagnoses** (of the diagnoses mentioned above) (diagnosis a + diagnosis b)		% of diagnosis b in diagnosis a	% of diagnosis a in diagnosis b
Migraine^**^ + TTH	670	67.5	62.6
Migraine + Chronic pain disorder with somatic and psychological factors (head)	200	20.1	36.9
Migraine^**^ + functional abdominal pain^††^	73	7.3	15.1
Migraine^**^ + back pain	12	1.2	11.3
TTH + functional abdominal pain^††^	101	9.4	20.8
TTH + back pain	15	1.4	14.2
functional abdominal pain^††^ + back pain	20	4.1	18.9

*Note*: ^*^ cumulative percentages exceed 100% due to multiple diagnoses per patient.

† e.g. medication overuse headache, posttraumatic headache.

‡e.g. headache unspecified.

§ e.g. abdominal pain unspecified.

|| e.g. back pain unspecified.

¶ e.g. spinal muscular athrophy, (rheumatoid) arthritis, Epidermolysis Bullosa, stump pain, phantom pain, muscular dystrophy, tumor, fractures, etc.

** including migraine without aura and with aura.

^††^ including functional abdominal pain, chronic pain disorder (abdomen) and other abdominal pain-related Functional Gastrointestinal Disorders.

In a striking number of children (55%) more than one pain diagnosis was detected. Migraine had a high co-morbidity with TTH and chronic pain disorder with somatic and psychological factors (head). TTH was also often diagnosed in patients with functional abdominal pain (Table 2).

**Figure 2:**
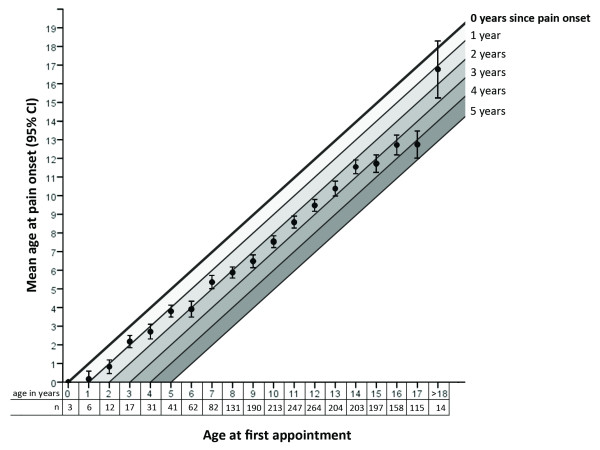
Relationship between age at pain onset and first appointment.

The median maximal pain intensity was 9.0 (NRS 0–10), median mean pain intensity 6.0 (Table 3). The pain values increased with increasing age (both *p’s* < 0.05; *eta^*2*^* ≥ 0.01). Girls’ pain ratings did not differ from boys (both *p’s ≥* 0.10). Thirty percent of children experienced pain several times per week and 43% reported daily or constant pain (Table 3). At the time of the initial assessment children had had pain for a mean of 31 months (Table 3); older children had pain for significantly longer than younger children (*F*(4,2180) = 19.45; *p* < 0.001; *eta^*2*^* = 0.03). Figure 2 shows the relation between age at pain onset and age at first appointment at the pain clinic. It indicates an increasing time to presentation the older the child at age of the initial presentation.

**Table 3:** Characteristics of pain, pain-related disability and emotional distress

**Item**	**N**	**Mean**	**SD**	**Min-Max**	**Percentiles**		
					**25%**	**50%**	**75%**
**Pain duration** (months)^*^	2190	31.1	32.2	1-193	7.0	19.0	45.0
**Maximal pain intensity** in the past four weeks (NRS 0–10)	2216	8.2	1.8	0-10	7.0	9.0	10.0
**Mean pain intensity** in the past four weeks (NRS 0–10)	2168	6.4	2.1	0-10	5.0	6.0	8.0
**Pain-related disability**^†^ (children > 3 years)	2083	36.0	10.8	12-60	29.0	36.0	43.0
**Anxiety**^‡^ (range 34–80) (children > 9 years)							
Fear of exam	1597	46.8	11.1	31-76	38.0	45.0	53.0
General anxiety	1595	50.3	11.4	34-80	41.0	49.0	57.0
School aversion	1598	51.2	10.8	35-79	41.0	50.0	57.0
**Depression**^§^ (range 33–80) (children > 8 years)	1621	51.6	10.9	33-80	44.0	50.0	59.0
**Frequency of pain episodes**		N = 2208		**n (%)**			
Constantly				635 (28.8)			
Daily				346 (15.7)			
Several times a week				662 (30.0)			
Less frequent				565 (25.6)			
**Days absent from school** in the past 4 weeks (children >6 years)		N = 1909					
No school absence / 1 day				925 (48.5)			
2–5 days				523 (27.4)			
> 5 days				461 (24.1)			

* a total of 156 children (6.9%) reported a pain duration of less than 3 month (this is independent of age group).

^†^ Paediatric Pain Disability Index (P-PDI [25]).

‡ Anxiety Questionnaire for Pupils [26].

§ Depression Inventory for Children and Adolescents [27].

### Pain-related disability, school absence and emotional distress

Nearly a quarter of the sample missed a substantial number of school days, 25% or more of their regular school days, due to pain although approximately half of the sample was not affected in their school attendance (≤ 1 day of school absence) (Table 3). The mean pain disability index was 36/60 (Table 3). Pain-related disability was higher in adolescents than younger children (*F*(3,2075) = 9.406; *p* < 0.001, *eta^*2*^* = 0.01). However, boys and girls were equally disabled (*F*(1,2075) = 0.004; *p* = 0.951). In this study 15% of children reported clinically relevant scores for fear of exam, 19% for general anxiety and 25% for school aversion. If these findings are compared to the general population, where 15% of the children obtain clinically relevant scores [26], then children with chronic pain had significantly more clinically relevant scores for general anxiety and school aversion (*Chi*^*2*^(1) = 17.555; *p* < 0.001; *Chi*^*2*^(1) = 121.442; *p* < 0.001). This was also reflected in the mean test score for school aversion (*t*(1620) = 4.282; *p* < 0.001), but not the mean test score for general anxiety (*t*(1620) = 1.001; *p* = 0.317) (Tables 3).

The number of children with clinically relevant depression scores was higher in the study group (24%) compared to the general population (15%) [27] (*Chi*^*2*^(1) = 101.518; *p* < 0.001). This was also reflected in the higher mean test score in comparison with the norm data (*t*(1620) = 5.998; *p* < 0.001) (Tables 3).

**Figure 3:**
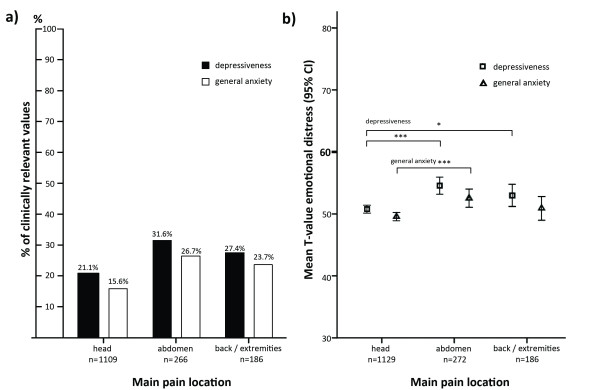
**Emotional distress of children by main pain locations.***Note.* Figure 3**a)** number of children with clinically relevant values in depression (*Chi^*2*^*(2) = 15.163; *p* < 001; Cramer’s V = .1) and general anxiety (*Chi^*2*^*(2) = 20.066; *p* < 001; Cramer’s V = .1); a T-value >60 is clinically relevant. Figure 3**b)** mean values of depression and anxiety scores; analyzed by ANOVA (Depression: *F*(2, 1584) = 13.977; *p* < 0.001; *eta^*2*^* = 0.02; General Anxiety: *F*(2, 1558) = 7.704; *p* < 0.001; *eta^*2*^* = 0.01). Results of post-hoc tests are depicted in the figure: * p < 0.05; ***p < 0.001.

Emotional distress differed between children depending on the location of pain. The number of children with increased values that were clinically relevant differed between pain locations (Depression: *Chi^*2*^*(2) = 15.163; *p* < 001; Cramer’s V = .1; General Anxiety *Chi^*2*^*(2) = 20.066; *p* < 001; Cramer’s V = .1). The amount of increased values was especially high in children with abdominal pain and lowest in headache patients (Figure 3a). A comparison of the mean scores of depression and anxiety revealed a significant difference between pain locations (Depression: *F*(2, 1584) = 13.977; *p* < 0.001; *eta^*2*^* = 0.02; General Anxiety: *F*(2, 1558) = 7.704; *p* < 0.001; *eta^*2*^* = 0.01) (Figure 3b), displaying the same pattern observed in clinically relevant values: children with abdominal pain showed higher scores for depression and general anxiety compared to children with headache. Emotional distress in headache patients was also significantly lower than in children with musculoskeletal pain.

### Health care utilization

Nearly 40% of the patients had a pain-related hospital stay before they sought treatment at the pain clinic (Table 4) with this being higher in children with musculoskeletal and abdominal pain compared to children with headache (*Chi^*2*^*(2) = 101.549; *p* < 0.001; Cramer’s V = .2). Children had on average 3.2 (*SD* = 3.1) previous physician consultations and diagnostic investigations with most children visiting a doctor 1 to 5 times and about 13% having had a very high number of previous treatments and consultations (≥ 6). In total, 90% of children reported taking analgesics before they presented to the clinic with 76% reporting medication use within the preceding 3 months (Table 4). In contrast, the number of children receiving a recommendation of analgesic treatment following their initial evaluation was 50% and of those children who had previously taken analgesics only 57% received a recommendation for further medication use.

**Table 4:** Health care utilization

**Item**	**n (%)**
**Pain motivated prior hospital stay (n = 2162)**	806 (37.3)
**Previous analgesic treatment (n = 2234)**	1999 (89.5)
Recommendation for treatment with analgesics after initial appointment (n = 1998)	1057 (52.9)
**Current analgesic treatment (n = 2235)**	1706 (76.3)
Recommendation for treatment with analgesics after initial appointment (n = 1705)	973 (57.1)
**Number of previous physician consultations and diagnostic investigations else than referring paediatrician (n = 2188)**	
0–5	1911 (87.3)
> 5	277 (12.7)

### Factors associated with extremely high impairment

**Figure 4:**
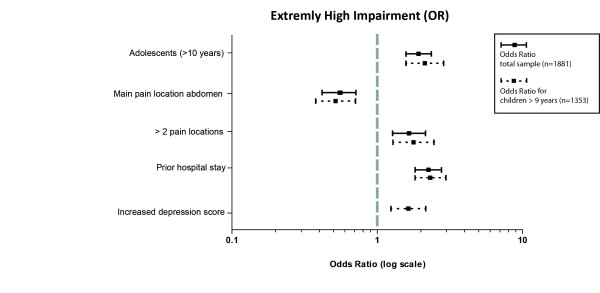
Factors associated with “extremely high impairment”.

There were 1043 of 1881 children (55%) assigned to the group of children with extremely high impairment. The sex of the child did not relate to the level of impairment but there was a significant association with the age of the child. Older children were at an increased risk of extremely high impairment. In addition, the factors multiple locations of pain, prior hospital stay, and increased depression scores were associated with impairment, while children with abdominal pain as the main location were less likely to be extremely high impaired (Figure 4).

## Discussion

This study showed that nearly 30% of children and adolescents with chronic pain presenting for tertiary care had constant pain and an alarming, median maximum pain intensity of 9 (NRS 0–10). Disturbing as this may be it is only the tip of the iceberg. The impact of the pain, like that previously found in adults [29], was prominent with children scoring highly for pain-related disability. Many children were no longer participating in normal every day activities, one quarter were missing more than 25% of school and emotional distress was high with clinically significant scores for depression (24%) and anxiety (19%).

The children in this study experienced a much higher negative impact from the pain with elevated levels of pain intensity [4] and pain frequency [30] in contrast to findings from epidemiology studies that included children with recurrent or permanent pain during the past 3 months. This study population also displayed significant interference in daily life, school attendance and emotional well-being. The presence of pain in different parts of the body has been previously illuminated [4,31,32] and this study delved further into the subject of co-morbid diagnoses, discovering a large number of children with a chronic pain disorder with somatic and psychological factors (43%) or more than one distinct pain diagnosis (55%). Additionally, children were experiencing elevated symptoms of anxiety and depression, 19% and 24% respectively.

Importantly, and as previously shown in other clinical and epidemiological findings [4,6,12]‐[15], the study population was dominated by female patients (61%) with this increase manifesting from the age of 13 years possibly suggesting an effect of pubertal development [33]. These findings, as previously suggested [4,6], underline adolescent girls as a very vulnerable group. Treatment studies also show that adolescent girls have a worse treatment outcome compared to boys [23,34]. Regarding pain location in this study, adolescent girls suffered from more musculoskeletal pain and pain in more than two locations. Sex differences with regards to pain location were not found in other age groups. This is in contrast to a recent German epidemiological study which investigated the location of the most bothersome pain. Girls reported this to be the head or the abdomen more often than boys [6]. Further studies in school children with chronic pain showed that girls experience more abdominal pain; boys more limb pain [4,5]. Sex difference in pain intensity found in school children with chronic pain, where girls report more intense pain compared to boys [4], could also not be replicated in our group of highly impaired children. In line with recent epidemiological findings [5], boys and girls in this sample did not differ in pain-related disability. Discrepancies between findings in epidemiological research and our sample can be interpreted in two ways: Either pain characteristics in highly impaired children with a chronic pain are different from school children who report the symptom of pain or differences are caused by a systematic effect of treatment seeking.

Over all, even though girls are more prominent in this tertiary care sample, group comparisons indicate age differences, rather than sex differences, as being implicated in pain severity and extremely high impairment in this sample. In this sample both pain intensity and pain-related disability increase with age. Epidemiological studies have not found these associations [4,5]. Arguably, the association between age and severity of pain, detected in this study of children with high impairment from chronic pain, was the most important finding. This may be partially explained by the worrying discovery that adolescents entering specialist management had had pain for a longer period of time, no matter the sex, compared to younger children. This kind of delay has the potential to increase developmental stasis, or even regression, in children of any age. Furthermore, extremely high levels of impairment were associated with previous hospital stays, increased depression and pain in several locations.

This level of complexity, along with the striking number of unsuccessful prior hospital stays would suggest problems with identification and referral of unsatisfactorily managed children and adolescents with chronic pain in the primary and secondary health care system. It is intended that patients seek treatment at a primary care level before calling upon tertiary treatment. Treatment in primary care will be sufficient in a large number of affected children. The elevated time since pain onset in our sample (median: 19 months, range: 1–193) may suggest that the referral to a pain specialist is carried out too late. With regards to our data, this interpretation would imply that this effect gets stronger the older the child; the average time from pain onset until receiving tertiary treatment in 15 year-old children is 4 years.

Since we did not control for pain severity in the past, an alternative interpretation of these findings could be developmental changes in the pain condition during adolescence. Adolescence is a vulnerable period of life. It may aggravate a preexisting pain problem and other associated problems (e.g. problems in school, emotional distress) thereby becoming a condition that requires treatment. Further research will be required to investigate this further.

Two diagnostic groups, in our study, that may highlight an insufficient knowledge in the primary care sector were adolescents with abdominal pain classified as chronic pain disorder with somatic and psychological factors (more than 10% of the children older than 10 years) and younger children diagnosed with migraine (48% of the children younger than 11 years). The prevalence of functional abdominal pain in the community is known to be more common in younger children [35] while prevalence data for migraine in the community suggests an increase with age [36]. Bhatia et al. [37] revealed a lack of knowledge of pediatricians and physicians in treating children with chronic pain in primary care which might be specifically prominent in these two groups. This lack of knowledge and earlier referrals to a tertiary pain service for children with more complicated diagnoses could be improved by a comprehensive advanced training program in chronic pain for primary pediatricians.

Improvement is not only needed in the primary care sector. Tertiary care structures for highly impaired children with chronic pain are scarce [1,2,17]. Children are often referred to specialized centers according to the location of pain i.e. to headache clinics [38,39], pediatric gastroenterologists [40] or institutions specialized in musculoskeletal pain [1]. However, this would seem inadequate given the findings that 55% of the children analyzed in this study have more than one distinct pain diagnosis partially affecting different parts of the body and nearly 20% of children reported pain in more than two locations.

The discrepancy between services offered and needed [2] makes planning for services based on demand unfeasible. One obvious reality stands out with the current situation; there is a lack of specialized clinics available to meet patient demand. It is of desperate interest to know why health care for this vulnerable group of children is so sparse and extensive investigation is crucial to identify the issues and, as a consequence rectify the situation.

Another worrying outcome of the study was the high number of children on analgesics (76%) without an evidence based indication for the analgesic used in 43%; that is a total of 33% of the sample taking analgesics without indication. This suggests medication use was inadequate for a sizable number of children. Misuse of analgesics may have detrimental effects such as the exacerbation of pain and disability caused by analgesic rebound headache following the overuse of analgesics in children with chronic headache [41].

Results of this study have to be seen in the light of some limitations. First, this study has the standard limitations of any retrospective analysis and any findings of significance can only reflect a correlation not a causal relationship. Second, the described sample aims to reflect the characteristics of children with a severe chronic pain condition, in keeping with referrals to a tertiary center. However, it is probable that not all children who could have benefited from involvement with the service during the study period were seen due to certain restrictions such as financial or distance barriers [42] or, indeed, inadequate referral patterns. And last, it has to be noted that parent proxy reports for pain characteristics were used in case of missing child report. Data for this study was gathered retrospectively from the clinical letters, where missing values from children are automatically replaced by parent reports. We examined the data of 529 children, who presented in the pain clinic between July 2009 and June 2010 (Wager et al., in prep.). In this subsample only 2% of missing child data on pain intensity was replaced by parent information.

## Conclusion

It is well known that chronic pain in children and adolescents is a serious problem with a wide range of consequences on the child’s development [1] and treatment needs to improve [37]. Regrettably, epidemiological data on children with chronic pain in need of specialized treatment does not exist with this study offering a first detailed analysis of children with chronic pain diagnoses requiring specialist intervention. The complexity of problems identified strongly suggests that children with chronic pain require pain clinics dedicated to the management of a wide spectrum of conditions to avert the considerable risk of developmental stagnation, misuse of analgesics and other associated problems of chronic pain. Care structures for highly impaired children with chronic pain still need improvement to guarantee the best possible treatment.

## Competing interests

The authors declare that they have no competing interests.

## Authors’ contributions

BZ, JW, TH, CH, UR, MD, BH, CW and MB contributed to the conception and design of the study. BZ, JW, AM and TH contributed to the analysis of data. All authors contributed to the interpretation of data, the drafting or revising of the manuscript, and final approval for publication. BZ and MB are the guarantors.

## Pre-publication history

The pre-publication history for this paper can be accessed here:

http://www.biomedcentral.com/1471-2431/12/54/prepub
